# Extraction Strategies to Recover Bioactive Compounds, Incorporation into Food and Health Benefits: Current Works and Future Challenges

**DOI:** 10.3390/foods9040393

**Published:** 2020-03-30

**Authors:** María del Mar Contreras, Eulogio Castro

**Affiliations:** 1 Department of Chemical, Environmental and Materials Engineering, Universidad de Jaén, Campus Las Lagunillas, 23071 Jaén, Spain; 2 Centre for Advanced Studies in Earth Sciences, Energy and Environment (CEACTEMA), Universidad de Jaén, Campus Las Lagunillas, 23071 Jaén, Spain

There are numerous studies in the literature about bioactive products (extracts, essential oils, oleoresins, hydrolysates, etc.), exhibiting numerous health benefits. Foods of plant and animal origin [1,2], medicinal plants [1,3], and marine products [4] can be their sources. We recognize the need to look for more efficient agroindustry because of unsustainable current practices, the population factor, and the state of natural resources. This requires us to move towards a more sustainable circular model that uses renewable resources, i.e., bioeconomy. Therefore, this implies a shifting to produce more food and to valorize agricultural byproducts and all agro-industrial derived streams through obtaining bio-based chemicals and products (including bioactive products), as well as bioenergy and/or biofuels [5]. To bring together new data to expand the information on bioactive compounds when applied to the food industry, we edited this special issue on “Extraction Strategies to Recover Bioactive Compounds, Incorporation into Food, and Health Benefits” (URL: https://www.mdpi.com/journal/foods/special_issues/extraction). 

One of the objectives of the special issue was to address new ways to extract bioactive compounds from raw materials, including agri-food waste, sustainably, and mainly using food-grade conditions. In this context, four articles have been accepted, applying several extraction strategies, mostly assisted by ultrasound, to recover and ascertain bioactive compounds in Ecuadorian fruits [6] and buckwheat flour [7], as well as in agro-industrial byproducts from the processing of pseudo-cereal food [7], sesame [8], and the olive oil industry [9]. In the first work, Martín-García et al. (2019) determined the free and bound phenolic compounds in dehulled whole buckwheat flour and three milling fractions: light flour, bran flour, and middling flour. For that, an ethanol-water solution was used to extract free phenolic compounds, being assisted by ultrasound, and diethyl ether/ethyl acetate was applied to recover bound phenolic compounds after an alkaline treatment of the residual fraction. The most abundant free phenolic compounds were rutin and epiafzelchin–epicatechin-*O*-dimethylgallate, whereas the most abundant bound phenolic compounds were catechin and epicatechin in all buckwheat flours. The highest content of bound phenolic compounds (around 0.7 g/kg) was found in middling and bran flours, highlighting that both entire byproducts fractions could be used to develop functional foods owing to the beneficial health benefits of buckwheat phenolic compounds. The work by Mekky et al. [8] gives new insight into the phenolic composition of sesame and in particular of the residual cake after oil extraction using reversed-phase high-performance liquid chromatography coupled to diode array detection and quadrupole-time-of-flight-mass spectrometry. The characterized compounds belonged to several classes, namely, hydroxybenzoic acids, hydroxycinnamic acids, flavonoids, and lignans. Their findings suggest that the antioxidant activity of the sesame cake extract was not only promoted by sesamol and lignans, which are the phenolic compounds previously reported in sesame, but *C*-glycosides and other compounds could also contribute to this bioactivity. Furthermore, Contreras and coworkers [9] have proposed an integrated scheme to recover oleuropein and other olive leaves antioxidants in the first extraction step assisted by ultrasound, proteins in a second extraction step along with mannitol and arabinose oligomers, and finally, a cellulose-enriched fraction was obtained residually, which can be subject to subsequent valorization (e.g., for obtaining biofuel). 

The extracts and fractions containing bioactive compounds can be addressed to formulate nutraceuticals, preservatives, such as antioxidant and antimicrobial additives, or functional ingredients for functional foods. Moreover, some plant foods can contain a high content of bioactive components, which can be predicted using mathematical models and based on color measurements, as showed Llerena and coworkers [6]. Yang et al. [10] directly obtained a resveratrol-enriched rice wine with enhanced antioxidant activity by adding *Polygonum cuspidatum* root powder, as a source of resveratrol, and fermentation. After 10 days of co-fermentation, rice wine with high levels of resveratrol (86 mg/L) was obtained. Ultrafiltration was also applied as an alternative treatment to boiling for the clarification and sterilization of the beverage while maintaining the bioactive components. 

In summary, several strategies have been applied to obtain bioactive products from different sources, including agro-industrial byproducts. We are pleased to present this special issue, which offers new opportunities for alternative strategies in food production, supply, and consumption to move into a more sustainable industry with zero-waste. However, further work is required to test their food applicability, for example, as antioxidant additives, while functional ingredients of particular importance are performing more in vivo and clinical studies to demonstrate bioactivity and functionality. Nonetheless, some of the products and extracts are based on phenolic compounds, whose bioactivity is highly recognized.
